# Sexual Inhibition and Sexual Excitation Scales in Men: Psychometric Properties of a Polish Adaptation

**DOI:** 10.1007/s10508-020-01837-1

**Published:** 2020-09-22

**Authors:** Krzysztof Nowosielski, Jacek Kurpisz, Robert Kowalczyk, Michał Lew-Starowicz

**Affiliations:** 1 Institute of Health Sciences, University of Opole, 45-060 Opole, Poland; 2 Department of Psychiatry, Pomeranian Medical University, Szczecin, Poland; 3 Department of Sexology, Andrzej Frycz Modrzewski Cracow University, Kraków, Poland; 4 Institute of Psychiatry, Centre of Postgraduate Medical Education, Warsaw, Poland

**Keywords:** Sexual behavior, Sexual inhibition, Sexual excitation, Psychometrics, Sexual risk, Hypersexuality

## Abstract

The present study aimed to develop a Polish version of the Sexual Inhibition/Sexual Excitation Scale (SIS/SES-PL) and explore its psychometric validity in a sample of 498 men aged between 18 and 55 years. We used exploratory and confirmatory factor analyses to obtain the best model fit. Out of the 45 items in the original scale, 32 items with eight subscales and three higher-order factors: (sexual excitation [SES], sexual inhibition due to performance failure [SIS1], and sexual inhibition due to performance consequences [SIS2]) were included in the SIS/SES-PL. The SIS/SES-PL was found to have a good and satisfactory fit (comparative fit index = .87; Tucker–Lewis Index = .85; root mean square error of approximation = .054; *χ*^2^ = 1108.7; *p *< .001; test–retest reliability Cronbach’s alpha = .93). A small correlation between age and the SIS1 and SIS2 scores was detected. However, forward multiple regression analysis revealed a significant correlation only between age and SIS1 [*β* = .23, *p *< .001, *R*^2^ for model = .05; *F*(1, 494) = 27.52, *p *< .001]. Furthermore, a moderate correlation between SIS1 and SES2, as well as SIS1 and general inhibition properties (measured using the Behavioral Inhibition Scale), was noted. In the case of SIS2, only small effects were observed, with the highest values for engaging in risky sexual behavior. Out of the total number of 42 variables, a moderate correlation between SES and the following was described: importance of sex, hypersexual behaviors, frequency of masturbation, frequency of sexual activities per month, sexual risk-taking, high promiscuity (measured using the Sociosexual Orientation Inventory Revised-Drive), general activation properties (measured using the Behavioral Activation Scale), neuroticism, and erotophilic tendencies (measured using the Sexual Opinion Survey-Short Form). The results of the study allowed us to conclude that the SIS/SES-PL might be a useful tool for tailoring therapy for men with sexual problems, as well as in the field of clinical research on sexual inhibition and excitation. Furthermore, it is a reliable and useful tool for measuring propensities for sexual excitement and inhibition.

**Electronic supplementary material:**

The online version of this article (10.1007/s10508-020-01837-1) contains supplementary material, which is available to authorized users.

## Introduction

According to the dual control model (DCM), the sexual functioning of men and women is a final result of cooperation of two neurobehavioral systems: inhibition and excitation. A delicate balance between these two systems in the brain is crucial for sexual responses independent of the type of stimuli (Bancroft & Janssen, 2000; Hodgson, Kukkonen, Binik, & Carrier, 2016; Kurpisz, Mak, Lew-Starowicz, Nowosielski, & Samochowiec, 2015)

For measurement of individual propensities of sexual excitation/inhibition, several questionnaires have been developed (Bancroft, Graham, Janssen, & Sanders, 2009; Kurpisz et al., 2015). The first psychometric tool was called the Sexual Inhibition/Sexual Excitation Scale (SIS/SES) and was intended for use in male individuals; it was later modified for use in both sexes. An increasing number of studies have shown that it is useful in measuring the levels of sexual excitation and inhibition and their correlates. For example, men scoring high on the SES (a scale measuring “general propensity for sexual arousal”) reported higher sexual satisfaction, whereas those scoring higher on the SIS1 (a scale measuring “inhibition proneness due to risks of failure in sexual performance” or “general inhibitory tone”) had an increased risk of sexual problems (dysfunction). Low scores on the SIS2 (a scale measuring “inhibition proneness due to threats related to consequences of sexual fulfilment” or “situational inhibition”) and high scores on the SES were associated with a greater probability of engagement in risky sexual behaviors (RSBs) (Bancroft & Janssen, 2000; Bancroft & Vukadinovic, 2004; Hodgson et al., 2016; Janssen & Bancroft, 2007; Janssen, Goodrich, Petrocelli, & Bancroft, 2009; Kurpisz et al., 2015; Rettenberger, Klein, & Briken, 2016; Velten, 2017; Walton, Cantor, Bhullar, & Lykins, 2017). Since the first publications, the SIS/SES scale has been used to assess sexual responsiveness in clinical (Bancroft & Janssen, 2001; Duits, van Oirschot, van Oostenbrugge, & van Lankveld, 2009; Louizos, McCann, & Knight, 2014) and nonclinical (Bancroft & Vukadinovic, 2004; Sarin, Amsel, & Binik, 2014) samples of men, showing score correlations with particular sexual functions. Thus, the SIS/SES results can be helpful in planning sex therapy for men with sexual difficulties or dysfunctions (Bancroft & Janssen, 2000; Kurpisz et al., 2015; Velten, 2017)

The SIS/SES has been translated into different versions and validated in a few studies: Portuguese, Italian, German, Spanish, Finish, and South Asian languages (Hindi, Urdu, Panjabi, Tamil, and Sinhalese-only linguistic validation) (Granados, Salinas, & Sierra, 2018; Malavige et al., 2013; Panzeri et al., 2008; Pinxten & Lievens, 2015; Quinta Gomes et al., 2018; Velten, Scholten, & Margraf, 2018). In most studies, the validated version was similar to the original version. However, in the Spanish version, 34 questions grouped in four main factor categories were included in the final analysis, with excellent model fit indices (Granados et al., 2018). Similarly, the Portuguese version revealed three higher-order factor models with 35 items and a similar model fit (Quinta Gomes et al., 2018). For this reason, linguistic validation and assessment of the psychometric properties using a Polish version of the SIS/SES (SIS/SES-PL) for men are needed to enable its use in daily clinical practice for counseling men with sexual dysfunctions. This study thus aimed to perform such analyses, and the following hypotheses were formed:

### **Hypothesis 1**

The Polish version will be different from the original scale, as with the Spanish and Portuguese versions (Granados et al., 2018; Quinta Gomes et al., 2018). However, we anticipate that a three higher-order model is needed to obtain the best fit indices (Carpenter, Janssen, Graham, Vorst, & Wicherts, 2008).

### **Hypothesis 2**

As in the original scale, there will be a moderate correlation between the SIS1 and SIS2 scores and age (Carpenter et al., 2008; Janssen et al., 2002). Furthermore, some correlations between the SIS/SES scores and RSBs, erectile function, premature ejaculation (PE), neuroticism, extraversion, level of restrictiveness to relationships, social desirability, general activation/inhibition propensity, and erotophilic tendencies (overlap) are expected (Carpenter et al., 2008; Janssen et al., 2002).

## Method

### Participants

The respondents were recruited between January 2016 and December 2017 via internet invitations posted on social media (Facebook) (online version), as well as among students of the Medical College in Sosnowiec and Pomeranian University in Szczecin (paper–pencil version). The eligibility criteria were physical and mental abilities to complete a questionnaire, age between 18 and 55 years, and ability to read in Polish. Participants with a history of depression or other severe mental disorders (including addiction to psychoactive substances), severe somatic diseases, thyroid dysfunction, myocardial infarction less than 6 months prior to the study, severe cardiovascular disorders (NYHA 3 and NYHA 4), and unstable coronary angina were excluded from this study. All respondents (*n* = 1720) were asked personally or invited via post on Facebook encouraging to participate in the study entitled “Sexual function of men in the context of dual control model” (the link for the online version of the questionnaire was provided- https://www.surveymonkey.com/r/RCSXPYZ) to complete the research questionnaire, which was preceded by a short description of the study protocol. Before enrollment to the study, all participants had to read the informed consent statement and agree to participate verbally (paper–pencil version) or by clicking “yes” in the online questionnaire. They were asked to answer the questionnaire on Day 0 and then between 2 and 8 weeks later. To identify the subjects in the re-test procedure, all respondents were asked to enter an anonymous and unique identification code when completing the questionnaire for the first time and after 2–8 weeks.

Out of 1720 men who entered in the online survey or were asked to complete the paper–pencil version, 15 did not agree to participate, and a further 866 dropped out after the first question. Among the 839 remaining respondents (440 in the online version and 399 in the paper–pencil version), 70 returned incomplete forms; 769 completed questionnaires were collected, and the response rate was 44.71%. Finally, 53 men scored above the threshold point of ≥ 11 on the Hospital Anxiety and Depression Scale (HADS), indicating depressive symptoms. As depression is a well-known risk factor of sexual dysfunction and might serve as a confounder (Hodgson et al., 2016), those individuals were excluded from the study; thus, the study population comprised 716 individuals. Furthermore, as the paper was designed to assess the validity of the SIS/SES in the population of heterosexual men, those identified themselves as homosexual, bisexual, or asexual (*n* = 121), as well as those who met the exclusion criteria and were suffering from severe comorbidities (*n* = 97), were excluded from the further analysis. The final study sample consisted of 498 respondents. Of them, 57 men participated in the re-test study and completed the questionnaire for the second time.

### Measures

The HADS was used for the sample exclusion/inclusion. Scores of < 8 were considered to indicate lack of depressive symptoms; scores of 8–10, borderline level for a depressive episode; and scores of ≥ 11, high risk of clinical depressive episode (Karakuła et al., 1996).

The study was based on a survey containing standard questions concerning sociodemographic data, religiosity, sexual history (including sexual orientation based on Kinsey scale findings), type and frequency of sexual activities, current relationship status, role of sex and satisfaction from sexual life, self-declared intravaginal ejaculation latency time (IELT), number of lifetime sexual partners, and reported presence (lifetime and in the last 6 months) of erectile dysfunction (ED), premature ejaculation (PE0), desire disorders (DDs), delayed ejaculation (DE), hypersexual behaviors, and distress. For ED, PE, DD, and DE, self-reports and the DSM-5 criteria were used (American Psychiatric Association, 2013). Sexual activity was defined as any of the following: vaginal, anal, or oral contact; caressing/cuddling; sexual foreplay; and masturbation.

RSBs were defined as one of the following: sexual contacts with more than one sexual partner at the same time; sexual activity with a casual person (one-night stand); multiple sexual partners; intercourse with an individual living with HIV; inconsistent use of condoms in oral, anal, and vaginal contacts; prostitution and use of services of an escort agency; sexual contacts under the influence of psychoactive substances (chemsex: drugs or alcohol); and drug injection using shared needles within the last 6 months (Nowosielski, Sipinski, Kuczerawy, Kozlowska-Rup, & Skrzypulec-Plinta, 2012).

Hypersexual behaviors were defied as a pattern of recurrent, intense, and excessive preoccupation with sexual fantasies, urges, and behavior that individuals struggle to control (Rettenberger et al., 2016; Walton et al., 2017).

The tendencies to react with sexual excitation and inhibition were measured using the SIS/SES-PL; the SIS/SES was originally developed by Janssen et al. (2002). This questionnaire consists of 45 items constituting three higher-order dimensions (factors): SES-20 items, sexual inhibition due to threats of performance failure (SIS1)-14 items, and sexual inhibition due to expected performance consequences (SIS2)-11 items. All questions have a four-point Likert scale coding (1 = strongly disagree to 4 = strongly agree), except for questions 17 and 45 (reverse coded). Higher scores reflect a higher propensity for inhibition or excitation. Each higher-order factor has subscales, i.e., SES: social interaction (nine items), visual stimuli (four items), fantasies (four items), and nonspecific stimuli (three items); SIS1: losing arousal easily (eight items), partner concerns (three items), and performance concerns (three items); SIS2: risk of being caught (four items), negative consequences of sex (three items), and pain/norms and values (four items). The internal consistency of the original scale was satisfactory-Cronbach’s alpha for the SES, SIS1, and SIS2 were .89, .81, and .73, respectively, and those for the test–retest were .76, .67, and .74 respectively (Janssen et al., 2002).

Male sexual function was measured using the Polish adaptation of the International Index of Erectile Function (IIEF), a 15-item scale assessing five domains of male sexual function: erectile function, orgasmic function, sexual desire, intercourse satisfaction, and overall satisfaction. Higher scores reflect better sexual functioning (Puchalski, Szymański, Kowalik, & Filipiak, 2013; Rosen et al., 1997). Additionally, the participants were asked to assess and provide their self-measured IELT (in minutes).

Personality traits were measured using the NEO-FFI Personality Inventory. This questionnaire consists of 60 self-rating items, rated on a five-point scale. These items form five traits: neuroticism, extraversion, openness to experience, agreeableness, and conscientiousness (Costa Jr. & McCrae, 2008; Zawadzki, 2007).

The Behavioral Inhibition/Behavioral Activation Scale (BIS/BAS) was used to examine the general activation/inhibition properties of individuals (Carver & White, 1994; Müller & Wytykowska, 2005). The BAS consists of three subscales: reward responsiveness, drive, and fun seeking. The BIS and BAS are widely used for assessing general inhibition and activation tendencies as functional systems in the brain.

The 11-item Polish version of the Sexual Sensation Seeking Scale (SSSS) was used to assess sexual adventurism. Response options are on a four-point Likert-type scale, ranging from 1-“not at all like me” to 4-“very much like me.” The higher score reflects sexual risk-taking and unsafe sexual behaviors (Janda, 1998; Kalichman et al., 1994).

The 9-question Sociosexual Orientation Inventory Revised (SOI-R) was used to measure individual restrictiveness to relationships. Low levels of restrictiveness are indicative of readiness to engage in uncommitted and multiple sexual relationships, while high levels are indicative of predisposition to long-term relationships (Jankowski, 2016; Penke & Asendorpf, 2008). This scale has three subscales: attitude, aggregating, and desire. Lower scores indicate high restrictiveness.

The five-item Sexual Opinion Survey-Short Form (SOS-SF) was used to measure affective and evaluative responses to sexual stimuli, i.e., level of erotophobia/erotophilia. Response options are from 1-“strongly agree” to 7-“strongly disagree.” Higher scores indicate more erotophobic tendencies (Janda, 1998; Rye, Glenn, & William, 2010).

The 29-item Social Desirability Questionnaire (SDQ) was used for the assessment of the tendency to respond in a socially desirable manner. The questionnaire was based on the Marlowe-Crown Social Desirability Scale and validated in a Polish population (Crowne & Marlowe, 1960; Drwal & Wilczyńska, 1980). The scale consists of 29 statement concerning personal attitudes and traits. Respondents are asked to read each item and decide whether the statement is “True” or “False” for her/him and correspond to her/his personality. Higher scores reflect conformity to social rules and conventions.

### Linguistic Validation

The authors of the original version of the SIS/SES were first asked for permission for translation and adaptation of the questionnaire by our team. Thereafter, a linguistic validation was executed in accordance with the MAPI Institute guidelines (Chassany, Sagnier, Marquis, Fullerton, & Aaronson, 2002). It was performed in the following order: forward translation, backward translation, pilot study, cognitive debriefing, and field tests.

The first two translations were conducted independently by a trained professional translator and the first author of this study. The questionnaire instruction, items, and response options were translated. After comparison of both translated papers, the first version of the SIS/SES-PL was constructed. Thereafter, backward translation into English was conducted by an independent bilingual translator. The second version of the SIS/SES-PL was designed after the translators compared the backward-translated version with the original English version. In the further phase, the questionnaire was completed by a group of 25 male students to test its clarity, appropriateness, intelligibility, and cultural relevance. The students were then interviewed to identify any difficulties in understanding or interpreting particular SIS/SES items. No major difficulties were noted. This final version of the SIS/SES was used for further psychometric analyses.

### Data Analysis

For the analyses, we used the Statistica 12.0 Pl computer software (StatSoft, Cracow, Poland) and IBM SPSS 20 computer software with AMOS (IBM SPSS Statistics for Windows, Armonk, NY: IBM Corp; 2012). All the variables were verified for missing values (less than 5%) and distribution normality (skewness and kurtosis). Values larger than 2 for skewness or larger than 7 for kurtosis were considered as indicative of nonnormality (West, Finch, & Curran, 1995). The significance level was set at *p *= .05.

#### Factor Analysis

A confirmatory factor analysis (CFA) was used to check the model fit using the AMOS for IBM SPSS 25. A CFI and a TLI of ≥ .95 and an RMSEA of ≤ .06 indicated a good model fit (Hu & Bentler, 1998; MacCallum, Browne, & Sugawara, 1996). If the CFI and RMSEA were unsatisfactory, modification indices were inspected to identify non-fitting items, and CFA was performed again to obtain the highest fit indices. An exploratory factor analysis and a principal component analysis (PCA) with promax rotation were performed to determine the factor structures of the original SIS/SES and its Polish version. Only the variables with a loading higher than .40 were included in the first-order model (Granados et al., 2018; Tabachnick & Fidell, 2007).

#### Reliability

To assess the reliability of the questionnaire, test–retest and internal consistency methods were performed. The test–retest reliability was defined using intraclass correlation coefficients (ICCs) (Nunnally & Bernstein, 1994). It was assumed that ICC values of > .40 reflect poor to fair agreement; .41–.60, moderate agreement; .61–.80, good agreement; and > .80, excellent agreement between the two measurements. Cronbach’s alpha coefficient was used to evaluate internal consistencies. Values of > .70 were considered to indicate adequate to excellent reliability (Bartko, 1966).

#### Construct Validity

Convergent and discriminant validities of the SIS/SES-PL were assessed on the basis of the correlations with other variables, which measure proximal and distal constructs using Pearson’s *r*. The SIS/SES-PL was checked for correlations with the following: SSSS, SOI-R, SOS-SF, SDQ, and BIS/BAS findings; number of sexual partners; frequency of vaginal sex and sexual activity; frequency of masturbation; engagement in RSBs; IELT; importance of sex to the respondent; satisfaction from sexual life; history of sexual dysfunction; presence of sexual dysfunction; and IIEF scores. *R* values of ≥ .10 were indicative of a small effect size; ≥ .30, medium; and ≥ .50, large (Cohen, 1992). Additionally, to assess the degree of the SIS/SES-PL correlation with age, forward multiple regression analysis (with age as the dependent variable) was conducted.

## Results

The majority of the respondents were living in cities, had secondary education, and were blue-collar workers (Table 1). Approximately 67.7% of the population was Catholic, whereas 24.8% were atheists. Regular participation in religious practices was reported by 27.9% of the sample. Most of the respondents reported to have some sexual problems in < 25% of sexual activities. The most frequent lifetime sexual problem was PE. Sexual dysfunction according to the DSM-5 criteria (based on questionnaire responses) was diagnosed in 12 men: 10 had PE, one had ED, and one had DE. The general characteristics of the population and the sexual behaviors are shown in Tables 1 and 2, respectively.

**Table 1:** General characteristics of the sample: quantitative variables

Variable	*M*	Median	Range	SD	Skewness	Kurtosis
Age (years)	31.85	28.00	18.59–65.00	11.17	1.20	0.57
Religiosity^a^	2.59	3.00	1.00–5.00	1.21	0.00	− 1.13
BMI	25.49	24.97	16.79–48.42	3.85	1.31	4.28
Age of first vaginal sex	18.19	18.00	14.00–32.00	2.73	1.54	3.99
Importance of sex^a^	3.80	4.00	1.00–5.00	0.76	− 0.11	− 0.33
Sexual life satisfaction^a^	4.05	4.00	1.00–5.00	0.83	− 0.79	0.65
Frequency of sexual activities a month	13.95	12.00	0.00–40.00	9.84	0.71	− 0.05
Number of lifetime sexual partners	5.33	4.00	0.00–25.00	4.91	1.61	2.53
Frequency of vaginal sex per month	8.80	8.00	0.00–30.00	6.92	0.92	0.30
Frequency of masturbation per month	5.37	4.00	0.00–25.00	6.09	1.32	1.21
Duration of the relationship	5.52	4.00	1.00–35.00	5.93	2.47	7.13
IELT (min)	14.64	15.00	0.00^b^–36.00	8.41	0.48	− 0.57
IIEF-EF	25.34	28.00	1.00–30.00	6.44	− 1.78	2.48
IIEF-IS	10.66	12.00	0.00–15.00	4.44	− 1.54	1.27
IIEF-OF	8.69	10.00	0.00–10.00	2.57	− 2.31	4.64
IIEF-SD	7.85	8.00	4.00–10.00	1.38	− 0.23	− 0.38
IIEF-OS	8.39	9.00	0.00–15.00	2.08	− 1.47	2.25
IIEF-total	61.07	66.00	7.00–75.00	13.91	− 1.75	2.43
NEO-FFI-neuroticism	19.99	20.00	0.00–44.00	7.71	0.29	− 0.05
NEO-FFI-extraversion	28.97	29.00	3.00–44.00	6.55	− 0.37	0.17
BAS-drive	11.03	11.00	5.00–16.00	2.56	0.17	− 0.47
BAS-fun seeking	11.81	12.00	4.00–16.00	2.25	− 0.29	0.24
BAS-reward responsiveness	15.23	15.00	5.00–20.00	2.36	− 0.42	1.44
BIS	18.94	19.00	10.00–28.00	3.37	− 0.10	0.22
SOS-SF	15.17	15.00	5.00–32.00	5.52	0.09	− 0.36
SDQ	12.33	12.00	3.00–25.00	4.21	0.33	− 0.28
SSSS	29.95	31.00	11.00–42.00	5.73	− 0.79	1.18
SOI-R-behavior	2.30	2.00	1.00–5.00	0.93	0.69	− 0.19
SOI-R-attitude	3.49	3.67	1.00–5.00	1.16	− 0.34	− 1.05
SOI-R-desire	2.93	3.00	1.00–5.00	1.13	0.06	− 0.93
SOI-R total	2.91	2.89	1.22–5.00	0.88	0.23	− 0.65

^a^Five-point Likert scale

^b^less than a minute (only two respondents stated that their IELT was 0 min)

IIEF, International Index of Erectile Function; EF, erectile function; IS, intercourse satisfaction; OF, orgasmic function; SD, sexual desire; OS, overall satisfaction; IELT, Intravaginal Ejaculation Latency Time; NEO-FFI, Big Five Personality Traits Inventory; BAS, Behavioral Activation Scale; BIS, Behavioral Inhibition Scale; SOS-SF, Sexual Opinion Survey, Short Form; SDQ, Social Desirability Questionnaire; SOI-R, Sociosexual Orientation Inventory Revised; SSSS, Sexual Sensation Seeking Scale

**Table 2:** Sociodemographic and sexological characteristics of the sample: quantitative variables

Variable	*n*	%
*Residency*		
Rural	83	16.8
City	412	83.2
*Education*		
Primary	95	19.1
Secondary	228	45.8
University	175	35.1
*Employment*		
White-collar worker	140	28.4
Blue-collar worker	248	50.4
Unemployed	86	17.4
Retired	19	3.8
Steady relationship	375	75.7
Sexual activity in last 12 months	484	97.1
RBS	123	24.7
ED-lifetime	149	29.9
PE-lifetime	220	44.2
Delay ejaculation-lifetime	205	41.2
Desire disorders-lifetime	121	24.3
Hypersexual behavior	130	26.2
Distress	104	21.6

RSB, risky sexual behavior; ED, erectile dysfunction; PE, premature ejaculation

The Kaiser–Meyer–Olkin (KMO) value confirmed the sampling adequacy for the analysis. The KMO value of .88 and Bartlett’s test of sphericity (*χ*^2^ = 7471.3; *p *< .001) indicated satisfactory correlations between the items. Initially, the CFA was performed using the assumption of the ten lower-factor model, three-factor model, and 10-in-3 higher-order model from the original validation (Janssen et al., 2002). The analysis revealed that the original models had unsatisfactory fit indices, with RMSEAs of .08, .06, and .61, respectively (Table 3). Thus, a Monte Carlo analysis was performed to determine the optimal number of statistically important lower-order factors for a new model (Wood, Akloubou Gnonhosou, & Bowling, 2015). Ten factors were extracted, with factors with eigenvalues > 1 accounting for 55.3% of the variances. Additionally, there were major differences in the items included to the particular subscales between the original and Polish versions. (Some new categories had to be created.) Furthermore, ten variables with loadings lower than .40 (original SIS/SES version questions 5, 8, 11, 14, 18, 25, 33, 38, 37, and 42) were eliminated from the model. As question 43 had a loading of > .40 in factor 4 and factor 8, it was also deleted. The ten identified factors/subscales were as follows: social interaction (Items 4, 6, 7, 13, 16, 26, 30, 32, 35, 39, and 44), partner characteristics (Items 17 and 45; previously included in the SIS1), visual stimuli (Items 1, 3, and 29), fantasies (Item 41), losing arousal easily (Items 9, 10, 36, and 40), circumstances (Item 2), performance concerns (Items 19–24), risk of being caught (Items 12 and 28), negative consequences of sex (Item 15), and pain/norms and values (Items 27, 31, and 34). The ten lower-factor model was found to show a poor fit in the CFA (RMSEA = .079) (Table 1). Thus, similar to the original validation, a higher-order analysis was then performed using principal axis with promax Kaiser normalization revealing three higher-order factors (KMO value = .73; Bartlett’s test of sphericity, *χ*^2^ = 989.3, *p *< .001) with eight subscales (two lower-order factors, i.e., negative consequences of sex and circumstances, were not entered into the model owing to their low loadings). The final model included 32 questions in three dimensions (8-in-3 factor model): SES with the subscales social interaction, partner characteristics, visual stimuli, and fantasies (Item 41) (17 items from the original model, eigenvalue = 2.63, percentage of explained variances = 26.3%); SIS1 with the subscales losing arousal easily, performance concerns, and risk of being caught (12 items from the original model, eigenvalue = 1.01, percentage of explained variances = 20.1%); and SIS2 with the subscale pain/norms and values (three items from the original model, eigenvalue = 1.01, percentage of explained variances = 20.1%). This model is accounted for 57.9% of the variance.

**Table 3:** Confirmatory factor analysis of the evaluated models

Model	*χ*^2^	df	CFI	NFI	TLI	RMSEA	% of explained variances (%)	KMO	Bartlett test	
									*df*	*χ*^2^
*Original model*										
Three factors	2803.7	942	.723	.637	.709	.063	30.8	.880	990	7471.3
Ten factors	3982.5	945	.549	.484	.527	.081	59.7	.880	990	7471.3
10-in-3 factors	2333.1	932	.747	.659	.731	0.61	64.0	.769	45	1539.3
*New model*										
Three factors	2143.7	663	.752	.678	.737	.067	35.3	.880	990	7471.3
Ten factors	1893.9	467	.683	.621	.664	.079	55.3	.880	990	7471.3
8-in-3 factors	1108.7	453	.870	.789	.852	.054	57.9	.727	45	989.3

CFI, comparative fit index; TLI, Tucker–Lewis index; RMSEA, root mean square error of approximation; KMO, Kaiser–Meyer–Olkin

**Fig. 1:**
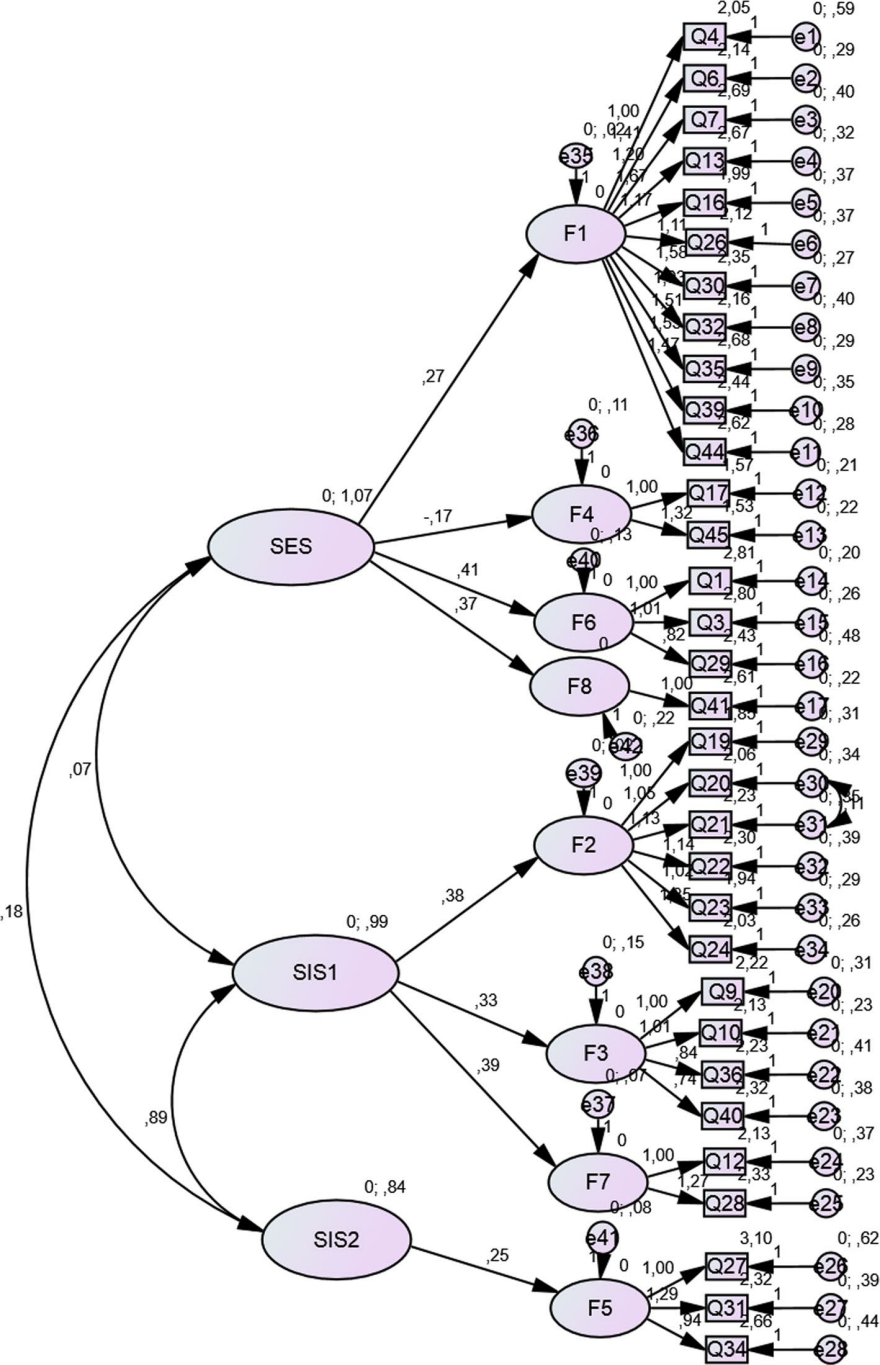
Final model of the Polish version of the sexual inhibition/sexual excitation scale with covariates and loadings

CFA was then performed on the new model (8-in-3 factor model). The initial analysis revealed moderate fit indices: CFI = .83; TLI = .82; RMSEA = .05; *χ*^2^ = 1180.0; *p *< .001. However, the modification indices suggested an improvement in the model fit when covariances between Items 20 and 21 (both items refer to sexual expectation during sexual activities) were detected and implied in the model. The final model reached a satisfactory fit: CFI = .87; TLI = .85; RMSEA = .054; *χ*^2^ = 1108.7; *p *< .001. Additionally, the three higher-order factors were not significantly inter-correlated (Fig. 1). As in the original validation study (Janssen et al., 2002), the three-factor model was also evaluated, and we performed similar analyses (PCA and CFA). This model did not reach a satisfactory fit; the indices for the analyses are shown in Table 3.

### Reliability

The SES and SIS1 demonstrated an excellent internal consistency as measured using Cronbach’s alpha (.86 and .84, respectively). However, the value in the SIS2 was low (.59). The test–retest reliability of the scale obtained after 2–8 weeks revealed a Cronbach’s alpha coefficient value of .93 (95% CI .86–.95) with a *p* value of < .0001. For the factors, the coefficients were as follows: SES, .89 (95% CI .82–.94); SIS1, .89 (95% CI .83–.94); and SIS2, .80 (95% CI .66–.89), showing an excellent test–retest reliability. The summary of the item analysis is shown in Table 4.

**Table 4:** SIS/SES-PL item analysis

Item	Mean	SD	Loading	*α* − 1	Item	Mean	SD	Loading	*α* − 1
SES	43.45	7.01	*α* − 0.86		SIS1	25.78	5.41	*α* − 0.84	
SES—mean for item—2.55 (1.98–3.47)					SIS1—mean for item—2.14 (1.85–2.33)				
1	2.81	0.71	1.86	0.86	9	2.22	0.76	1.00	0.84
3	2.80	0.76	1.86	0.86	10	2.13	0.70	0.98	0.84
4	2.05	0.83	1.49	0.87	12	2.13	0.78	1.06	0.84
6	2.14	0.70	2.10	0.86	19	1.85	0.69	1.19	0.83
7	2.69	0.74	1.81	0.86	20	2.06	0.72	1.27	0.83
13	2.67	0.77	2.49	0.85	21	2.23	0.75	1.36	0.83
16	1.99	0.71	1.75	0.86	22	2.30	0.77	1.28	0.84
17	3.43	0.59	0.76	0.87	23	1.94	0.68	1.21	0.83
26	2.12	0.70	1.68	0.86	24	2.03	0.74	1.43	0.83
29	2.43	0.83	1.77	0.86	28	2.33	0.78	1.24	0.83
30	2.35	0.72	2.37	0.85	36	2.23	0.77	1.15	0.84
32	2.16	0.71	1.55	0.86	40	2.32	0.73	1.00	0.84
35	2.68	0.72	2.24	0.85	SIS2	8.08	1.69	*α* − 0.49	
39	2.44	0.76	2.28	0.86	SIS2—mean for item—2.69 (2.31–3.10)				
41	2.61	0.76	1.69	0.86	27	3.10	0.87	1.05	0.41
44	2.62	0.70	2.21	0.85	31	2.32	0.78	1.44	0.41
45	3.47	0.68	1.00	0.87	34	2.66	0.75	1.00	0.37

*α* − 1—Cronbach’s alpha when the item is deleted

SIS, sexual inhibition scale; SES, sexual excitation scale; SIS/SES-PL, Polish version of the SIS/SES

### Score Distribution

The analysis of the skewness and kurtosis of the SES, SIS1, and SIS2 scores revealed distribution normality (skewness and kurtosis for the SES, SIS1, and SIS2 scores: .12 and .39; .22 and .35; and .03 and .74, respectively).

### Construct Validity

The convergent and discriminant validities of the SIS/SES-PL were assessed using Pearson’s *r* product-moment coefficient or Yule’s phi in the case of dichotomous variables (Table 5).

**Table 5:** Bivariate correlations of the SIS/SES-PL score with other variables

Variable	SIS1	SIS2	SES
Age (years)	.23^d^	.14^d^	−.04
Religiosity^a^	.01	−.02	−.23^d^
Importance of sex^a^	−.19^d^	−.07	.25^d^
Sexual life satisfaction^a^	−.14^d^	−.05	.02
Steady relationship	−.03	.11^c^	−.07
Age of first vaginal sex	.04	.01	−.01
IELT (min)	−.14^d^	−.06	.08
IIEF-total	−.17^d^	−.01	.16^d^
ED-lifetime	.27^d^	.11^*^	−.01
ED-last 6 months^b^	.25^d^	.08	−.10^c^
ED-DSM-5	−.06	−.08	−.01
PE-lifetime	.14^d^	.04	−.00
PE-last 6 months^b^	.09	.06	−.07
PE-DSM-5	.02	−.01	.02
Delayed ejaculation-lifetime	.02	.00	−.04
Delayed ejaculation-last 6 months^b^	.03	−.02	−.07
Delay ejaculation-DSM-5	.00	−.05	−.06
Desire disorders-lifetime	−.02	.01	.04
Desire disorders-last 6 months^b^	.11	.03	−.11
Desire disorders-DSM-5	.05	.04	.01
Distress	.19^d^	.01	.04
Hypersexual behaviors	−.06	−.09^*^	.29^d^
RBS	−.1^d^	−.16^d^	.13^d^
Nr of lifetime sexual partners	.01	−.13^d^	.16^d^
Frequency of vaginal sex a month	−.03	−.09^c^	.09^c^
Frequency of masturbation a month	−.09^c^	−.05	.32^d^
Frequency of sexual activities a month	−.19^d^	−.12^d^	.17^d^
NEO-FFI-neuroticism	.21^d^	−.04	.18^d^
NEO-FFI-extraversion	−.11	−.11	−.02
BAS-drive	−.05	−.08	.26^d^
BAS-fun seeking	−.05	−.08	.20^d^
BAS-reward responsiveness	−.06	−.05	.37^d^
BIS	.37^d^	.17^c^	.24^d^
SOS-SF	.10	.14^c^	−.41^d^
SDQ	−.03	.05	−.00
SSSS	−.12	−.06	.24^d^
SOI-R-behavior	.01	−.10	.16^c^
SOI-R-attitude	−.14	−.10	.26^d^
SOI-R-desire	−.04	−.07	.44^d^
SOI-R total	−.07	−.11	.36^d^
SIS1	–	.34^d^	.07
SIS2	.34^d^	–	.07
SES	.07	.07	–

^a^Five-point Likert scale

^b^Five-point Likert scale: in < 25%, 25–50%, 50%, 50–75%, and > 75% of the cases

^c^*p *< .05

^d^*p *< .001

IELT, Intravaginal Ejaculation Latency Time; IIEF, International Index of Erectile Function; RSB, risky sexual behavior; ED, erectile dysfunction; PE, premature ejaculation; NEO-FFI, Big Five Personality Traits Inventory; BAS, Behavioral Activation Scale; BIS, Behavioral Inhibition Scale; SOS-SF, Sexual Opinion Survey-Short Form; SDQ, Social Desirability Questionnaire; SOI-R, Sociosexual Orientation Inventory Revised; SSSS, Sexual Sensation Seeking Scale; SIS, Sexual Inhibition Scale; SES, Sexual Excitation Scale; SIS/SES-PL, Polish version of the SIS/SES

As expected, a medium correlation was detected between the SIS1 and SIS2 scores. A small correlation between age and the SIS1 and SIS2 scores was detected. However, forward multiple regression analysis revealed a significant correlation only between age and the SIS1 score [*β* = .23, *p *< .001, *R*^2^ for model = .05; *F*(1, 494) = 27.52), *p *< .001].

As assumed, the SES score was moderately correlated with the importance of sex, hypersexual behaviors, frequency of masturbation, frequency of sexual activities per month, sexual risk-taking (RSBs and SSSS findings), high promiscuity (SOI-R-Drive), general activation properties (BAS findings), neuroticism, and gerontophilic tendencies (SOS-SF findings). Furthermore, the SIS score was correlated with the presence of ED, PE, and distress (Table 5).

## Discussion

This study aimed to examine and validate the SIS/SES-PL. The statistical analysis enabled the creation of a version consisting of 32 questions, three main dimensions, and eight subscales. Thus, the first hypothesis in the present study was confirmed: the SIS/SES-PL needed few modifications in comparison with the original version, which was developed for an American population. Although the analysis allowed extraction of eight subscales in the three-dimensional model (one excitation and two inhibition factors), 13 of the 45 original items were deleted from the questionnaire to meet the requirements for a good model fit. Similar modifications were needed during the validation process of the SIS/SES in other languages such as Dutch (Janssen et al., 2002), German (Velten et al., 2018), Portuguese (Quinta Gomes et al., 2018), and Spanish (Granados et al., 2018). Surprisingly, in the Polish validation, Items 17 and 45 of the SIS1 in the original version were moved to the partner characteristics subscale of the SES. This might be attributed to cultural differences: women might be more open to sexual encounters in relationships and are thus more likely to be aroused, inducing excitement in the male partner (Nowosielski, Wrobel, & Kowalczyk, 2016). The SIS2 was reduced to three items. Some items from SES2 were transferred to SIS1, while others were excluded. For example, Items 12 and 28 (which are related to the risk of being caught) were transferred, as these items were concerned more with failure than consequences, i.e., whether or not individuals are willing to have sex outdoors without fear of being seen; Item 8 (which asks about fear of unwanted pregnancy) was deleted as this is more a concern among women than among men; Item 18 (which concerns sex without condom) was excluded as both unwanted pregnancy and STIs are still not considered a major threat in Poland and the use of a condom might be considered as lack of trust in a steady relationship; and Items 22 and 24 (which are related to being caught while masturbating and being heard while having sex) were moved to the performance concern subscale of SIS1, as these events might not be perceived as embarrassing, i.e., inhibition in different mechanisms (Pastwa-Wojciechowska & Izdebski, 2014). Finally, only three items (Items 31 and 34-related to pain as a consequence of sexual activity and item 27-having sexually transmitted infection) constituted the SIS2.

It seems that for every language and cultural milieu, the same statements on attaining or inhibiting sexual responses can be understood differently. Conversely, it does not exclude an important role of the specific context of the described reaction; taboos concerning sexuality (e.g., masturbation and pornography use); type of relationship, in which it is socially accepted to have sex; contraception use habits; level of sexual education; and tendency to give a sexual meaning to the personally experienced arousal in the contact with stimulus (which can be based on feeling of guilt and defensive mechanism of denial). Thus, there is no universal set of situations, which can be equally exciting or inhibiting for men from various countries. Notably, despite all the efforts and attention put into the proper translation procedure, the final version of the SIS/SES-PL may have some linguistic imperfections, which seem to be inevitable in every translation process (Malavige et al., 2013).

The study on the Polish validation of the SIS/SES has confirmed that sexual excitation and inhibition are independent systems. Similar to previous reports (Bancroft et al., 2009), there was no significant correlation between the scores of the SES, SIS1, and SIS2, while the relationship between the two inhibition scale scores was moderate. Based on the Cronbach’s alpha score, the obtained reliability for the SES and SIS1 was satisfactory; however, that for the SIS2 was relatively low. In other SIS/SES validation studies, the SES was found to be the most reliable scale, while the SIS2 was found to always have the lowest alpha score (Granados et al., 2018; Janssen et al., 2002; Pinxten & Lievens, 2015; van Lankveld, Platteau, van Montfort, Nieuwenhuijs, & Syroit, 2015).

To evaluate the second hypothesis, the selected variables and their relationships with the SES, SIS1, and SIS2 scores were analyzed. The propensity for sexual excitation was not related to age, while the inhibition scale scores were found to increase with age. Other studies showed various relationships between the SIS/SES scores and age: decreased SES score, increased SIS1 and/or SIS2 score, or both (Bancroft et al., 2009; Bancroft & Janssen, 2000; Pinxten & Lievens, 2015).

Erotophilia was found to be moderately correlated with higher SES scores and lower SIS2 scores. Similar relationships for the SES score were found in other studies(Bancroft, 2009; Wilson, Holm, Bishop, & Borowiak, 2002). It was noted that the SES score was significantly correlated with diminished restrictiveness and tendency to have multiple sexual partners, more frequent sexual fantasies, or feelings of desire for other individuals who are not currently their sexual partners (a weak to moderate correlation with all of the scores of the SOI-R subscales-behavior, attitude, and desire, as well as its total score). The strongest relationship was found between the scores of the SES and the desire subscale. However, there was no significant association with any of the inhibition scale scores. This shows the relevance of the SES in assessing spontaneous excitement responses to sexual stimulus and its important role in decision-making regarding engagement in less conservative or socially accepted sexual behaviors.

There were no significant correlations between all of the SIS/SES scores and social desirability level. Janssen et al. (2002) reported similar findings. Weak relationships between both sexual functions and behaviors and propensity for sexual excitation were noted. The total IIEF score was associated positively with the SES score and negatively with the SIS1 score. These findings indicate that erectile functions are not simply a reflection of sexual excitation and/or inhibition. The relationship between both tendencies resulting in sexual responses is more complex than the functions measured using the IIEF. Notably, the SIS/SES does not contain items related to orgasm, which could weaken the correlation. As for sexual behaviors, the respondents with higher SIS1 scores reported a shorter IELT. No significant correlations with other scale scores were found.

The propensity for sexual inhibition, especially on the SIS1, was found to be significantly correlated with sexual dysfunctions. This was associated with ED (both lifetime and during the last 6 months) and PE (lifetime). Higher SIS2 scores were associated with lifetime erectile problems (however, the effect was small), while lower SES scores were related to ED in the last 6 months. Desire disorders and delayed ejaculation were not significantly related to excitation or inhibition propensity. No significant associations between the SIS/SES scores and the DSM-5 diagnostic criteria for male sexual dysfunctions were found.

Risky sexual behaviors (Bancroft et al., 2009; Bancroft & Vukadinovic, 2004; Janssen & Bancroft, 2007; Turchik, Garske, Probst, & Irvin, 2010) and their correlation with the SIS/SES-PL scores were analyzed. Our study showed that the respondents who admitted to engage in risky sexual behaviors had higher SES scores and lower SIS2 scores, while those engaging in RSBs had higher scores in the SES and lower scores in both inhibition scales. The control of sexual inhibition/activation balance seems to be an important part, but is only a fragment of the entire process of compulsive sexual/hypersexual behavior. Conversely, a tendency for sexual sensation seeking (propensity to achieve optimal levels of arousal and engage in new sexual experiences) was associated only with higher scores in the SES, but not an inhibition tendency. Similar findings were reported elsewhere (Gaither & Sellbom, 2003; Nguyen et al., 2012).

To have a broader overview on the dual control of the sexual response system, a few psychological characteristics were measured, i.e., personality traits and general behavioral activation and inhibition systems. In previous studies concerning personality traits, the SES, SIS1, and SIS2 scores were found to be correlated with neuroticism (Bancroft et al., 2009). According to the Big Five Personality Traits theory, there was an association between neuroticism and the SES and SIS1 scores in this study. Although a positive correlation with the SIS1 score was expected, there was also a positive relationship found with the SES score, in contrast to previous reports. Such an observation can be explained by the characteristics of the studied sample, as well as the ambiguous role of negative emotions (especially anxiety) in the regulation of sexual responses (Bancroft, 2009). Anxiety and anger can be both exciting and inhibiting for different individuals. This paradoxical situation can be explained by the “excitation transfer” of the arousal rooted in the cognitive interpretation of the fearful stimulus (from “causing avoidance” to “exciting”). Probably, the studied sample was more vulnerable to react in this manner in the context of stable personality traits.

To meet the validation requirements, the sexual response system was compared with the more general system of behavioral control according to Gray’s theory or the approach-avoidance theory, and the BIS/BAS questionnaire was used (Janssen et al., 2002). As was expected, the SES score was associated with higher BAS scores on all of the subscales, with the strongest correlation found with RR, as well as higher behavioral inhibition tendencies. Such an outcome is consistent with previous findings (Janssen et al., 2002). The BIS score was also moderately related to the SIS1 score and related to the SIS2 score. (However, the effect was small.) No other significant correlations were noted. However, in previous studies, negative correlations between the SIS1 and BAS (reward responsiveness and fun seeking) scores and between the SIS2 and BAS (fun seeking) scores were observed.

The present study had some limitations. First, further validation processes, especially in homosexual and bisexual individuals, are required. Second, as individuals with depressive symptoms were excluded from the study, the relationship between the SIS/SES-PL score and depression/anxiety should be analyzed in further research. Third, the pencil–paper version should be compared with the online version. Despite the aforementioned limitations, we believe that our study has important contributions regarding the DCM. It also has a major clinical implication; as the scale was properly validated, it can be used as a tool in tailoring therapy for sexual dysfunctions in men.

## Electronic supplementary material

Below is the link to the electronic supplementary material.

Supplementary material 1 (DOC 78 kb)
