# Supplementary material for: Sexual Inhibition and Sexual Excitation Scales in Men: Psychometric Properties of a Polish Adaptation
Source: Arch Sex Behav. 2020 Sep 22;50(6):2741–53. doi: 10.1007/s10508-020-01837-1 (PMC8416830; doi:10.1007/s10508-020-01837-1)
Supplement: Supplementary file 1 — Supplementary material 1 (DOC 78 kb) [file 10508_2020_1837_MOESM1_ESM.doc]

**Skala Pobudzenia i Hamowania Seksualnego
wersja polska – SIS/SES-PL ©**

**Instrukcja.**

W kwestionariuszu znajdzie Pan stwierdzenia dotyczące tego, jak mógłby Pan zareagować podczas różnych sytuacji, aktywności lub zachowań o charakterze seksualnym.
Pana reakcje mogą być oczywiście uzależnione od różnych okoliczności. Z tego powodu, wypełniając kwestionariusz proszę zaznaczyć takie odpowiedzi, które najtrafniej opisują Pana reakcję w podanych sytuacjach.

Proszę uważnie przeczytać każde stwierdzenie i zaznaczyć, w jaki sposób by Pan zareagował w podanych sytuacjach. Wybraną odpowiedź proszę zakreślić kółkiem.

Proszę postarać się odpowiedzieć na wszystkie stwierdzenia.

W niektórych pytaniach, może Pan odnieść wrażenie, że żadna z odpowiedzi nie opisuje Pana reakcji. Również niektóre stwierdzenia mogą Pana nie dotyczyć. W tych sytuacjach proszę wybrać taką odpowiedź, która opisywałaby Pana możliwą reakcję, gdyby Pan znalazł się w opisywanej sytuacji.

W wielu stwierdzeniach znajdzie Pan takie określenia jak: „podniecony seksualnie” lub „podniecony”. Poprzez te określenia należy rozumieć „uczucie seksualnego podniecenia”, odczucie „stymulacji seksualnej”, bycia „napalonym”, „seksualnie nakręconym”. Określenie „erekcja” stosowane w wielu stwierdzeniach, oznacza wzwód członka związany z podnieceniem seksualnym.

Proszę nie zastanawiać się zbyt długo nad odpowiedziami i zaznaczać swoje pierwsze skojarzenie.

Prosimy nie pomijać żadnego stwierdzenia. Prosimy także o bycie możliwie najbardziej szczerym w udzielanych odpowiedziach

©J. Bancroft, E. Janssen

Polska walidacja: Nowosielski K, Kurpisz J, Kowalczyk R, Lew-Starowicz M.

|  |  | **Zdecydowanie  się nie zgadzam** | **Nie zgadzam się** | **Zgadzam się** | **Zdecydowanie  się zgadzam** |
| --- | --- | --- | --- | --- | --- |
|  |  |  |  |  |  |
| 1 | Kiedy patrzę na erotyczne zdjęcie, łatwo się podniecam. | 1 | 2 | 3 | 4 |
|  |  |  |  |  |  |
| 2 | Kiedy samotnie oglądam sceny erotyczne w filmie, szybko się podniecam. | 1 | 2 | 3 | 4 |
|  |  |  |  |  |  |
| 3 | Czasem podniecam się leżąc po prostu na słońcu. | 1 | 2 | 3 | 4 |
|  |  |  |  |  |  |
| 4 | Kiedy nieznana mi, atrakcyjna seksualne osoba przypadkowo mnie dotknie, łatwo się podniecam. | 1 | 2 | 3 | 4 |
|  |  |  |  |  |  |
| 5 | Podczas intymnej kolacji przy świecach z osobą atrakcyjną dla mnie seksualnie, podniecam się. | 1 | 2 | 3 | 4 |
|  |  |  |  |  |  |
| 6 | Abym mógł utrzymać erekcję, mój członek musi być dotykany. | 1 | 2 | 3 | 4 |
|  |  |  |  |  |  |
| 7 | Podczas seksu, muszę skupić się na moich uczuciach seksualnych, by utrzymać erekcję. | 1 | 2 | 3 | 4 |
|  |  |  |  |  |  |
| 8 | Jeśli kocham się w ustronnym miejscu poza domem, ale wydaje mi się, że ktoś jest w pobliżu, jest mało prawdopodobne, bym się bardzo podniecił. | 1 | 2 | 3 | 4 |
|  |  |  |  |  |  |
| 9 | Kiedy widzę kogoś atrakcyjnego w seksownym ubraniu, łatwo się podniecam. | 1 | 2 | 3 | 4 |
|  |  |  |  |  |  |
| 10 | Kiedy rozmawiam przez telefon z osobą o seksownym głosie, podniecam się. | 1 | 2 | 3 | 4 |
|  |  |  |  |  |  |
| 11 | Kiedy widzę, że partner/ka jest podniecona seksualnie, moje podniecenie wzrasta. | 1 | 2 | 3 | 4 |
|  |  |  |  |  |  |
| 12 | Nie potrafię się podniecić, chyba że skupiam się jedynie na bodźcach seksualnych. | 1 | 2 | 3 | 4 |
|  |  |  |  |  |  |
| 13 | Jeśli wiem, że ktoś oczekuje, że się podniecę, mam problemy z uzyskaniem podniecenia. | 1 | 2 | 3 | 4 |
|  |  |  |  |  |  |
| 14 | Jeśli martwi mnie to, że mogę nie zaspokoić seksualnie partnera/partnerki łatwo tracę erekcję. | 1 | 2 | 3 | 4 |
|  |  |  |  |  |  |
| 15 | Kiedy się masturbuję i przeczuwam, że ktoś w każdej chwili może wejść do pokoju, stracę erekcję. | 1 | 2 | 3 | 4 |
|  |  |  |  |  |  |
| 16 | Jest mi trudno podniecić się, chyba że fantazjuję o wyjątkowo podniecającej sytuacji. | 1 | 2 | 3 | 4 |
|  |  | **Zdecydowanie  się nie zgadzam** | **Nie zgadzam się** | **Zgadzam się** | **Zdecydowanie  się zgadzam** |
|  |  |  |  |  |  |
| 17 | Jeśli inni mogą usłyszeć mnie podczas seksu, jest mało prawdopodobne, bym był dalej podniecony. | 1 | 2 | 3 | 4 |
|  |  |  |  |  |  |
| 18 | Kiedy biorę kąpiel lub prysznic, łatwo się podniecam. | 1 | 2 | 3 | 4 |
|  |  |  |  |  |  |
| 19 | Jeśli uświadomię sobie, że mogę zarazić się chorobą weneryczną, jest mało prawdopodobne, bym był dalej podniecony. | 1 | 2 | 3 | 4 |
|  |  |  |  |  |  |
| 20 | Jeśli ktoś może mnie zobaczyć, kiedy się kocham, jest mało prawdopodobne, bym był dalej podniecony. | 1 | 2 | 3 | 4 |
|  |  |  |  |  |  |
| 21 | Kiedy z innymi oglądam film pornograficzny, szybko się podniecam. | 1 | 2 | 3 | 4 |
|  |  |  |  |  |  |
| 22 | Kiedy nieznana mi, atrakcyjna seksualnie osoba patrzy mi prosto w oczy, podniecam się. | 1 | 2 | 3 | 4 |
|  |  |  |  |  |  |
| 23 | Jeśli pomyślę, że seks sprawi mi ból, tracę erekcję. | 1 | 2 | 3 | 4 |
|  |  |  |  |  |  |
| 24 | Kiedy mam na sobie coś, w czym czuję się atrakcyjny seksualnie, to jest prawdopodobne, że się podniecę. | 1 | 2 | 3 | 4 |
|  |  |  |  |  |  |
| 25 | Jeśli seks miałby sprawić ból partnerowi/partnerce, to jest mało prawdopodobne, bym był dalej podniecony. | 1 | 2 | 3 | 4 |
|  |  |  |  |  |  |
| 26 | Kiedy myślę o bardzo atrakcyjnej osobie, łatwo się podniecam. | 1 | 2 | 3 | 4 |
|  |  |  |  |  |  |
| 27 | Kiedy mam już erekcję, chcę jak najszybciej rozpocząć współżycie, by jej nie stracić. | 1 | 2 | 3 | 4 |
|  |  |  |  |  |  |
| 28 | Kiedy widzę atrakcyjną osobę, zaczynam wyobrażać sobie, jak się z nią kocham. | 1 | 2 | 3 | 4 |
|  |  |  |  |  |  |
| 29 | Kiedy myślę o innych rzeczach, łatwo tracę erekcję. | 1 | 2 | 3 | 4 |
|  |  |  |  |  |  |
| 30 | Fantazje seksualne często pomagają mi utrzymać erekcję. | 1 | 2 | 3 | 4 |
|  |  |  |  |  |  |
| 31 | Kiedy flirtuje ze mną atrakcyjna osoba, łatwo się podniecam. | 1 | 2 | 3 | 4 |
|  |  |  |  |  |  |
| 32 | Zaspokajanie partnerka/partnerki podczas seksu sprawia, że jeszcze bardziej się podniecam. | 1 | 2 | 3 | 4 |

**Scoring remarks. .**

SES score is calculated by adding items: 1,2,3,4,5,9,10,11,18,21,22,24,26,28,30, 31 and 32

SIS1 score is calculated by adding items: 6,7,8,12,13,14,15,16,17,20,27, and 29.

SIS2 score is calculated by adding items: 19,23 and 25.

SIS (general score) = SIS1 + SIS2.

Higher score reflects higher propensity.
